# The interplay of gut microbiota, obesity, and depression: insights and interventions

**DOI:** 10.1007/s00018-024-05476-w

**Published:** 2024-10-30

**Authors:** Iryna Halabitska, Pavlo Petakh, Iryna Kamyshna, Valentyn Oksenych, Denis E. Kainov, Oleksandr Kamyshnyi

**Affiliations:** 1 Department of Therapy and Family Medicine, I. Horbachevsky Ternopil National Medical University, Voli Square, 1, Ternopil, 46001 Ukraine; 2 Department of Biochemistry and Pharmacology, Uzhhorod National University, Uzhhorod, 88000 Ukraine; 3 Department of Medical Rehabilitation, I. Horbachevsky Ternopil National Medical University, Ternopil, 46001 Ukraine; 4 Department of Clinical Science, University of Bergen, Bergen, 5020 Norway; 5 Department of Clinical and Molecular Medicine (IKOM), Norwegian University of Science and Technology, Trondheim, 7028 Norway; 6 Department of Microbiology, Virology, and Immunology, I. Horbachevsky Ternopil National Medical University, Ternopil, 46001 Ukraine

**Keywords:** Obesity, Depression, Gut microbiota, Mental health, Antidepressants, Probiotics

## Abstract

The gut microbiome, body weight, and related comorbidities are intricately linked through a complex interaction of microbial, genetic, environmental, and psychological factors. Alterations in gut microbiota can contribute to the development of weight disorders and depressive symptoms, with the potential for these relationships to be bidirectional. Effective management of these interconnected conditions often involves a combination of lifestyle modifications and psychological support. Medical interventions, including treatments for obesity, antidiabetic drugs, antidepressants, antibiotics, and probiotics, can have beneficial and detrimental effects on gut microbiota and mental health. Further research is needed to better understand their impact on gut microbiome and mental health in the context of obesity.

**Supplementary Information:**

The online version contains supplementary material available at 10.1007/s00018-024-05476-w.

## Introduction

Genetic predispositions, socio-demographic variables, environmental conditions, psychological states, lifestyle choices, infections, and medication use influence the interplay between microbiome and health (Fig. S1a). Disruptions in the gut microbiota can lead to metabolic and psychological dysregulation, contributing to obesity and depression through mechanisms such as chronic inflammation, altered metabolism, and hormonal imbalances (Fig. S1b). Understanding these interactions is crucial for advancing our health knowledge and developing targeted prevention and treatment strategies for associated diseases [1, 2]. This review examines the interconnections between gut microbiota, obesity, and depression.

## Intestinal microbiota

The gut microbiota, composed of over 1,000 species of bacteria, archaea, fungi, protozoa, and viruses significantly impacts human health [3, 4]. It plays a crucial role in immune system maturation, maintaining intestinal integrity, producing vitamins K and B, preventing infections, and regulating metabolism [5].

The bacterial genome content in the gut is 150 times larger than the human genome [4]. The main microbial phyla in the gut are *Actinobacteria*,* Bacteroidetes*,* Firmicutes*,* Fusobacteria*,* Proteobacteria*, and *Verrucomicrobia*, with *Bacteroidetes* and *Firmicutes* making up about 90% of the gut microbiome [6, 7]. A balanced gut microbiota, or eubiosis, enhances immunity, nutrient absorption, blood glucose and lipid balance, and energy metabolism, while also producing essential vitamins and protecting against pathogens. Imbalanced gut microbiota, or dysbiosis, can result from antibiotics, poor diet, stress, and other factors, leading to health issues [8, 9].

Gut microbiota composition varies across the gastrointestinal tract due to differences in environment and flow rates. Factors like age, diet, antibiotics, and exercise influence the microbiome. Gut bacteria metabolize dietary polysaccharides into short-chain fatty acids (SCFAs), providing about 70% of the energy for colon cells and contributing to glucose homeostasis, lipid metabolism, immune function, and overall health [10].

## Obesity

Obesity, affecting over 650 million people globally, significantly contributes to morbidity and mortality [11]. Defined by a body mass index (BMI) over 30 kg/m2 (overweight is 25–30 kg/m2), obesity results from an imbalance between energy intake and expenditure, leading to excessive fat accumulation. It is linked to diseases like type 2 diabetes, cardiovascular diseases, certain cancers, and high blood pressure [12].

This complex disease is influenced by diet, antibiotic use, environment, genetics, lifestyle, socioeconomic factors, and gut microbiota composition. High-calorie diets contribute to obesity and alter gut microbiome function. Modern lifestyle factors, hormonal imbalances, and genetic influences drive the obesity crisis. Gut hormones communicate food intake and energy stores to the brain, regulating appetite [13].

## Depression

Depression is a common chronic mental disorder characterized by symptoms such as insomnia, melancholy, lack of enjoyment in life, and low energy. Its prevalence is estimated at 11% in low- to middle-income countries and 15% in high-income countries, affecting about 280 million people globally [14, 15]. Annually, depression and anxiety cost an estimated US$ 1 trillion due to the loss of 12 billion productive workdays [16].

Depression is significantly correlated with various physical health conditions, including suicide, cancers, respiratory diseases, diabetes, and cardiovascular diseases. In the USA alone, depression-related suicides claim about 40,000 lives annually, among older men [17]. According to WHO reports from 2016, around 785,000 suicides occur worldwide each year, with up to 60% attributed to depression [18, 19].

## Interplay between gut microbiota, obesity and depression

**Fig. 1:**
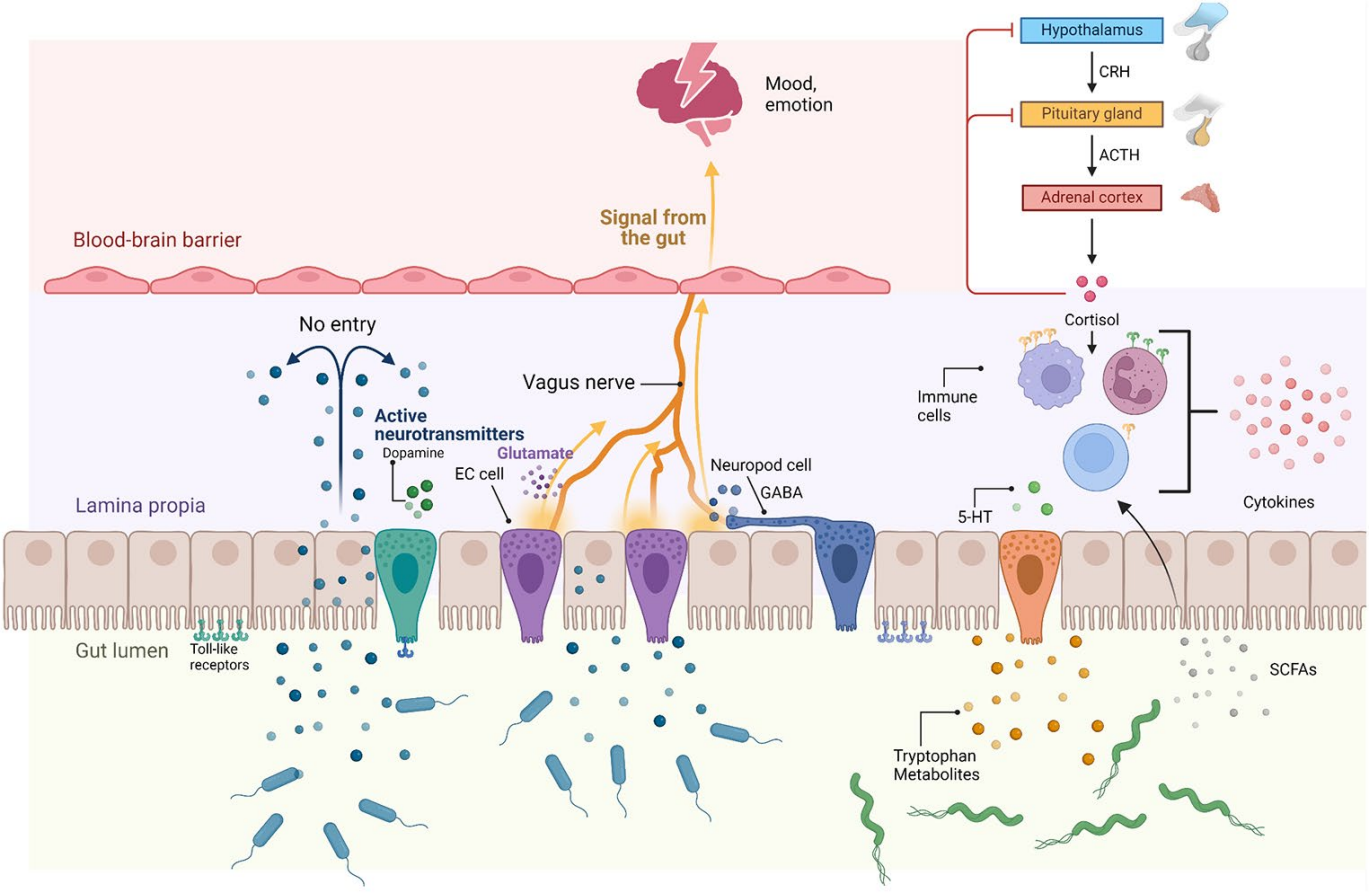
Gut-brain axis signaling pathways and their influence on mood and emotion regulation. The diagram depicts the interactions between the gut lumen, gut microbiota, and the central nervous system through the vagus nerve, immune cells, and neurotransmitter signaling. Various gut-derived signals, including active neurotransmitters (dopamine, serotonin, glutamate, and gamma-aminobutyric acid (GABA)), as well as tryptophan metabolites and short-chain fatty acids (SCFAs), influence mood and emotional regulation by transmitting signals to the brain. This communication occurs through the vagus nerve, immune modulation, and the hypothalamic-pituitary-adrenal (HPA) axis, which involves the secretion of corticotropin-releasing hormone (CRH), adrenocorticotropic hormone (ACTH), and cortisol. Dysregulation in these pathways can impact mood and emotional states, contributing to stress, depression, and anxiety

There is a link between gut microbiome, obesity, and depression (Fig. 1). For example, an increased *Firmicutes/Bacteroidetes* ratio is associated with obesity. However, this ratio alone cannot fully explain obesity due to the gut microbiome’s complexity [20, 21]. For instance, mutations in the leptin gene in mice have been linked to altered bacterial proportions [8, 20]. The gut microbiome affects energy intake and metabolism, influencing weight and fat storage [22, 23].

**Fig. 2:**
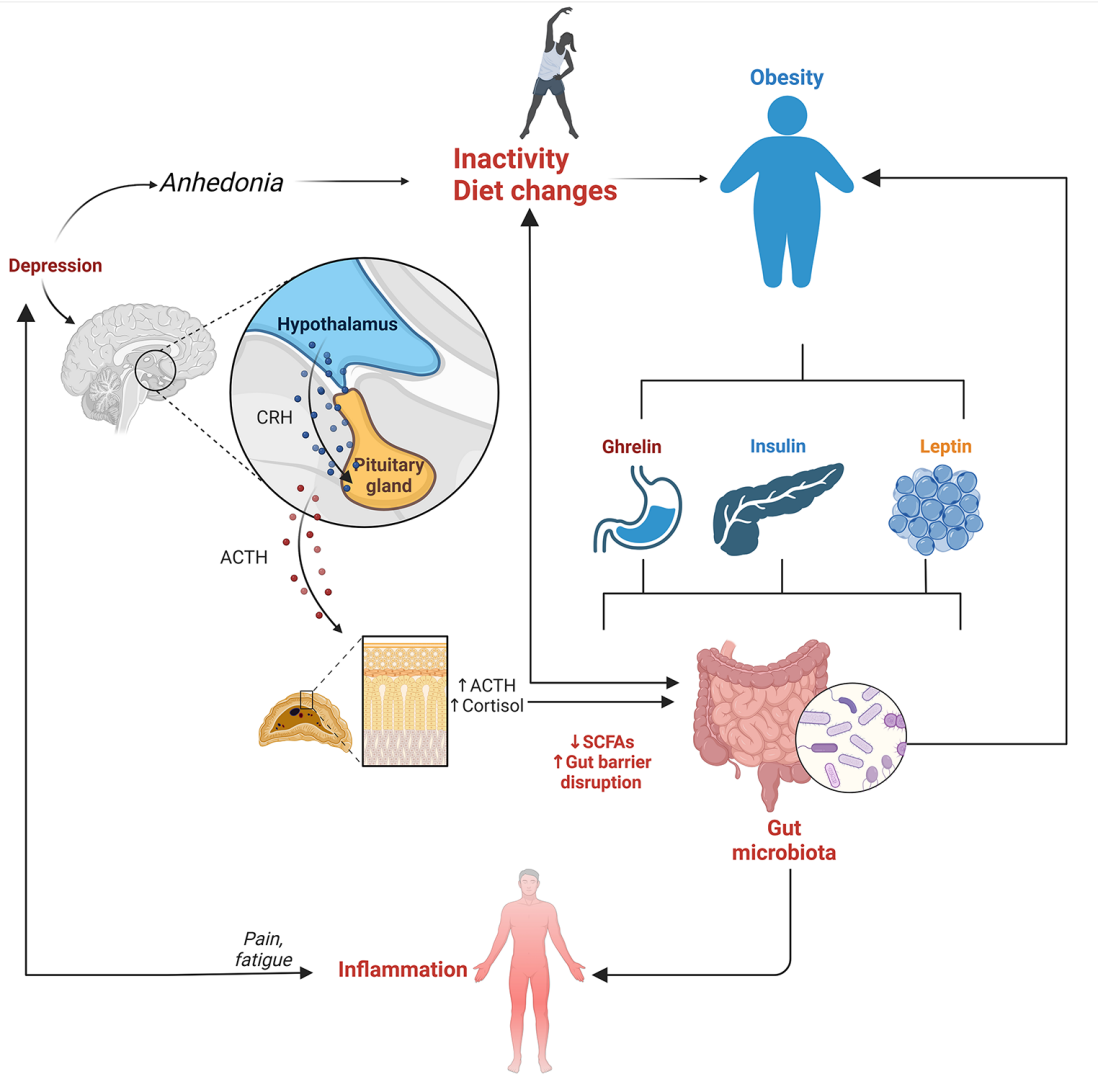
Interconnection between obesity diet inactivity gut microbiota and the hypothalamic-pituitary-adrenal (HPA) axis contributing to inflammation and depression. The diagram illustrates the complex relationship between inactivity and diet changes, leading to obesity, which interacts with hormonal regulators like ghrelin, insulin, and leptin. These factors influence gut microbiota, leading to reduced short-chain fatty acids (SCFAs) and increased gut barrier disruption. Such disturbances in gut health elevate cortisol and adrenocorticotropic hormone (ACTH) levels through the activation of the HPA axis, fueling inflammation, pain, fatigue, and depressive symptoms, including anhedonia. This bidirectional feedback loop highlights the role of chronic inflammation in exacerbating psychological and metabolic health issues

Obese individuals often have microbiomes that increase energy extraction and fat accumulation. Gut microbiome composition, such as higher *Prevotella* levels, is associated with greater weight loss compared to *Bacteroides* dominance [24, 25]. Inflammation caused by microbiota can affect leptin expression and thermogenesis, contributing to obesity. Chronic low-grade inflammation, driven by lipopolysaccharides (LPS), impairs energy expenditure (Fig. 2) [9].

The gut microbiome also impacts vitamin synthesis, xenobiotic metabolism, and obesity regulation. Studies using germ-free mice have highlighted the microbiome’s role in energy metabolism and mental health. The gut-brain axis connects microbiome composition with brain function and mental disorders, such as depression [26–28]. Previous studies indicate alterations in the gut microbiota of patients with mental disorders, with the most studied phyla being *Firmicutes*,* Bacteroidetes*,* Actinobacteria*,* Proteobacteria*, and *Verrucomicrobia*, and key genera including *Prevotella*,* Coprococcus*,* Parabacteroides*,* Phascolarctobacterium*,* Escherichia-Shigella*,* Bacteroides*,* Alistipes*, and *Veillonella* [29].

Specific bacteria, like *Alistipes* and *Veillonella*, are associated with depression, while others, like *Prevotella*, are less prevalent [27, 30]. Dysbiosis, including imbalances in gut microbiota, is linked to depression and systemic inflammation. Microbial lactate accumulation may also impair brain function and contribute to mental disorders [31, 32].

The link between gut microbiota and mental health, particularly depression, is increasingly recognized through the concept of the gut-brain axis. This bidirectional communication pathway allows gut bacteria to influence brain function via neurotransmitter production, immune responses, and signaling molecules. Disruptions in gut microbiota, or dysbiosis, can lead to imbalances in neurotransmitters like serotonin, increase systemic inflammation, and impair gut barrier function, all of which may contribute to depressive symptoms [28]. Dysbiosis can lead to an increase in pro-inflammatory cytokines and a disruption in the synthesis of key neurotransmitters like serotonin, dopamine, and gamma-aminobutyric acid (GABA), contributing to the development of metabolic and mood disorders [33].

Obesity is often associated with mental health issues, particularly depression. High body weight is linked to poorer mental health due to systemic inflammation, hypothalamic-pituitary-adrenal axis dysregulation, and psychological stress from weight stigma and discrimination, exacerbating mental health problems and hindering weight control efforts [34].

Furthermore, imbalances in gut microbiota can impair the production of crucial metabolites like SCFAs, disrupting gut-brain signaling and leading to dysregulation of the hypothalamic-pituitary-adrenal (HPA) axis. This dysregulation can result in elevated cortisol levels and altered serotonin production, which are associated with stress, anxiety, and mood disorders [35–37].

Obesity is influenced by genetics, metabolism, and environmental factors, with growing evidence highlighting the role of gut microbiota in these interactions. Dysbiosis in the gut microbiome has been linked to obesity through pathways that affect metabolism, insulin resistance, and satiety, offering potential therapeutic targets for prevention and treatment [38, 39].

## Interventions of gut microbiota, obesity and depression

Lifestyle modifications, including diet, stress management, exercise, and avoiding harmful substances, are commonly recommended for managing altered gut microbiota, obesity, and depression (Table S1). While these strategies can improve quality of life, long-term results are often modest, with only 61% of individuals completing such programs successfully. In severe cases, medical interventions, surgical options, and psychotherapy may be needed to address these complex issues (Table S2, S3). Effective management can enhance health outcomes and reduce the prevalence of related conditions like type 2 diabetes, cardiovascular disease, and certain cancers, thereby improving overall public health [40].

### Antibiotics and probiotics

Antibiotics like amoxicillin and ciprofloxacin can disrupt gut microbiota by killing both harmful and beneficial bacteria. This imbalance may lead to altered neurotransmitter levels, increased inflammation, and gastrointestinal issues, potentially contributing to mood disturbances and depressive symptoms.

Probiotics, such as *Lactobacillus rhamnosus* and *Bifidobacterium longum*, can help restore a balanced gut microbiota. They may improve mental health by enhancing gut barrier function, modulating neurotransmitter levels, and reducing stress and anxiety. These effects vary by strain and individual response, but these probiotics are commonly studied for their potential mental health benefits [41, 42].

### GLP-1 receptor agonists

In 2016, the American Association of Clinical Endocrinology reviewed five anti-obesity medications (orlistat, lorcaserin, naltrexone–bupropion, liraglutide, and phentermine–topiramate) for long-term use. However, the FDA withdrew lorcaserin in 2020 due to cancer risks. In 2021, studies on semaglutide, a new weekly Glucagon-Like Peptide-1 (GLP-1) receptor agonist, showed promising results that could impact clinical practice. Other diabetes medications, such as Sodium-Glucose Cotransporter 2 (SGLT2) inhibitors, metformin, and pramlintide, also show potential for obesity management [43, 44].

**Fig. 3:**
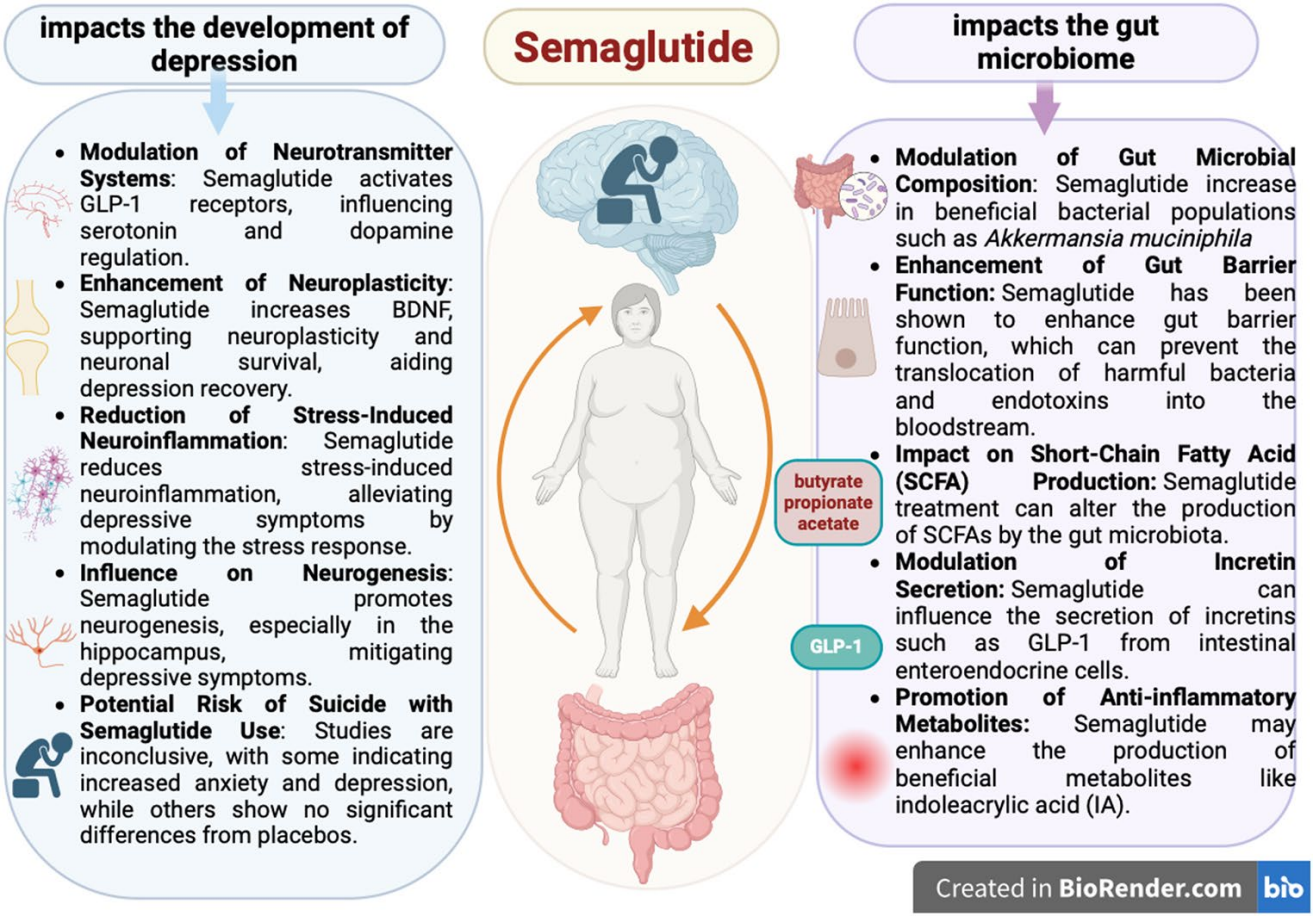
Effect of semaglutide on mental health and gut microbiome. This diagram illustrates how Semaglutide influences mental health and changes the gut microbiota, showing its combined effects on managing obesity and related conditions

Anti-obesity drugs like liraglutide and semaglutide affect gut microbiota, enhancing bacterial diversity and influencing metabolic disorders. Semaglutide significantly alters gut microbiota, impacting mental health and metabolic pathways. Liraglutide has shown therapeutic effects on fatty liver and modifies gut bacteria linked to metabolism and inflammation (Fig. 3) [45–47].

Both liraglutide and semaglutide impact the gut microbiota in conditions like Polycystic Ovary Syndrome (PCOS). They influence the abundance of various bacterial species and promote beneficial bacteria, altering the gut microbiome’s diversity and composition. These changes may optimize obesity treatment outcomes [46].

Studies on GLP-1 receptor agonists reveal mixed effects on mental health. Semaglutide and liraglutide have been associated with depressive symptoms, anxiety, and, in some cases, suicidal thoughts. However, there’s no clear causal link between GLP-1R agonism and severe depression or suicide. Monitoring for mood changes is advised for patients using these medications [48–50].

Recent social media discussions also reflect concerns about GLP-1 receptor agonists affecting mood and mental health, indicating the need for ongoing vigilance and assessment [51].

### Antidiabetic drugs

Metformin affects gut microbiota by altering its composition, enhancing SCFA-producing bacteria like *Butyricimonas* and beneficial probiotics such as *Lactobacillus*, while reducing harmful pathogens like *Prevotella* [52, 53]. It improves glucose control in obese mice and enhances SCFA-producing bacteria, and in T2DM and COVID-19 patients, it boosts beneficial bacteria like *Bacteroides* while lowering harmful ones [54]. Metformin might also impact mental health and depressive symptoms, potentially offering benefits for treatment-resistant depression. However, the exact mechanisms of its effects on mental health need further research [55, 56].

### Anti-obesity pharmacological agents

Naltrexone-Bupropion has been shown to influence gut microbiome diversity [57]. Orlistat treatment results in a reduction of *Firmicutes* and an increase in *Bacteroidetes*, with associated improvements in the diversity indices and elevated levels of *Lactobacillus*, particularly *Lactobacillus gasseri* [58]. Topiramate increases *Lactobacillus johnsonii* but significantly reduces the overall abundance of *Lactobacillus* [59]. SGLT2 Inhibitors alter the prevalence of specific bacteria, including the LPS-producing *Oscillibacter* and SCFA-producing *Bacteroides* and *Odoribacter*, while boosting *Ruminococci*, which are beneficial for SCFA production [60].

Regarding mental health outcomes, behavioral weight loss (BWL) and Naltrexone-Bupropion have demonstrated efficacy in treating binge-eating disorder, with BWL showing superior results compared to no intervention [61]. Orlistat has been linked to increased physical activity and reduced depression in Latinx patients with obesity, although these effects were not directly associated with weight loss [62]. Phentermine-Topiramate has been shown to alleviate depressive symptoms in obese patients and improve quality of life, though it poses risks such as hypomania in bipolar patients and potential psychosis when combined with venlafaxine [63].

SGLT2 inhibitors have improved quality of life in diabetes patients and show promise for adjunctive treatment of major depressive disorder (MDD) [64]. These findings highlight the complex relationship between weight loss medications, gut microbiota changes, and psychiatric outcomes.

### Antidepressants

Antidepressants are pharmacological agents used primarily to treat major depressive disorder (MDD) and other mood disorders by modulating neurotransmitter systems in the brain. The most widely prescribed antidepressants include Selective Serotonin Reuptake Inhibitors (SSRIs), such as fluoxetine and sertraline, which enhance serotonergic neurotransmission by inhibiting the reuptake of serotonin. Serotonin-Norepinephrine Reuptake Inhibitors (SNRIs), including venlafaxine and duloxetine, act on both serotonin and norepinephrine, aiming to alleviate depressive symptoms through dual neurotransmitter modulation. Tricyclic Antidepressants (TCAs) and Monoamine Oxidase Inhibitors (MAOIs), though less commonly used as first-line treatments due to their broader side effect profiles, remain effective for treatment-resistant cases and specific mood disorders. TCAs, such as amitriptyline, affect multiple neurotransmitter systems, while MAOIs, such as phenelzine, inhibit monoamine oxidase, leading to increased levels of serotonin, norepinephrine, and dopamine [65, 66].

Atypical antidepressants, including bupropion and mirtazapine, exhibit diverse mechanisms and are employed based on individual patient profiles and specific clinical needs. Bupropion, for instance, primarily affects norepinephrine and dopamine systems, while mirtazapine enhances neurotransmitter release through its antagonistic effects on certain receptors. The therapeutic efficacy of antidepressants is often accompanied by a range of side effects, which vary by drug class and individual patient response. Monitoring and managing these effects are crucial, as abrupt discontinuation of antidepressants can precipitate withdrawal symptoms. Overall, the choice of antidepressant and treatment regimen should be tailored to the patient’s specific condition and response to therapy, with ongoing assessment to optimize outcomes and minimize adverse effects [65, 66].

The studies demonstrate that fluoxetine significantly affected the growth of *E. coli*, *E. faecalis*, and *S. aureus*, with minimal impact on *C. albicans*. In contrast, escitalopram notably influenced the growth of *E. coli*, *E. faecalis*, *B. bifidum*, *L. rhamnosus*, and *C. albicans*, while mirtazapine exhibited the greatest activity against *L. rhamnosus* and *C. albicans* [67]. Antidepressants can modify the abundance and composition of intestinal microbiota, which in turn affects treatment outcomes for depression by influencing drug metabolism, absorption, and blood-brain barrier permeability [68].

## Conclusions

Changes in gut microbiota and the presence of depressive disorders are commonly noted in obesity, with these conditions potentially aggravating each other. The influence of different treatments—such as antibiotics, probiotics, anti-obesity medications, antidiabetic agents, and antidepressants—on gut microbiota and mental health is often intricate and multifaceted. These interventions can produce conflicting effects and may differ considerably among individuals. Consequently, thorough research is required to clarify how these treatments affect gut microbiota and mental health.

## Electronic supplementary material

Below is the link to the electronic supplementary material.

Supplementary Material 1

## Data Availability

All data generated or analyzed during this study are included in this published article and its supplementary information files.

## References

[1] Carmody RN, Bisanz JE (2023) Roles of the gut microbiome in weight management. Nat Rev Microbiol 21(8):535–550 37138047 10.1038/s41579-023-00888-0 PMC13306846

[2] Gou W, Miao Z, Deng K, Zheng JS (2023) Nutri-Microbiome epidemiology, an emerging field to disentangle the interplay between nutrition and microbiome for human health. Protein Cell 14(11):787–806 37099800 10.1093/procel/pwad023 PMC10636640

[3] Behzadi P, Dodero VI, Golubnitschaja O (2024) Systemic inflammation as the Health-Related Communication Tool between the Human Host and gut microbiota in the Framework of Predictive, Preventive, and Personalized Medicine. All around Suboptimal Health: Advanced approaches by Predictive, Preventive and Personalised Medicine for healthy populations. Springer, pp 203–241

[4] Lathakumari RH, Vajravelu LK, Satheesan A, Ravi S, Thulukanam J (2024) Antibiotics and the gut microbiome: understanding the impact on human health. Med Microecology. :100106

[5] Mukherjee S, Patra R, Behzadi P, Masotti A, Paolini A, Sarshar M (2023) Toll-like receptor-guided therapeutic intervention of human cancers: molecular and immunological perspectives. Front Immunol 14:1244345 37822929 10.3389/fimmu.2023.1244345 PMC10562563

[6] Rinninella E, Raoul P, Cintoni M, Franceschi F, Miggiano GAD, Gasbarrini A, Mele MC (2019) What is the healthy gut microbiota composition? A changing ecosystem across age, environment, diet, and diseases. Microorganisms 7(1):14 30634578 10.3390/microorganisms7010014 PMC6351938

[7] Behzadi P, Najafi A, Behzadi E, Ranjbar R (2016) Microarray long oligo probe designing for Escherichia coli: an in-silico DNA marker extraction. Cent Eur J Urol 69(1):105 10.5173/ceju.2016.654 PMC4846717 27123336

[8] Muscogiuri G, Cantone E, Cassarano S, Tuccinardi D, Barrea L, Savastano S, Colao A (2019) Obesity programs of nutrition E, Research, group A. Gut microbiota: a new path to treat obesity. Int J Obes Supplements 9(1):10–19 10.1038/s41367-019-0011-7 PMC6683132 31391921

[9] Montenegro J, Armet AM, Willing BP, Deehan EC, Fassini PG, Mota JF, Walter J, Prado CM (2023) Exploring the influence of gut microbiome on energy metabolism in humans. Adv Nutr 14(4):840–857 37031749 10.1016/j.advnut.2023.03.015 PMC10334151

[10] Li Z, Yi C-X, Katiraei S, Kooijman S, Zhou E, Chung CK, Gao Y, van den Heuvel JK, Meijer OC, Berbée JF (2018) Butyrate reduces appetite and activates brown adipose tissue via the gut-brain neural circuit. Gut 67(7):1269–1279 29101261 10.1136/gutjnl-2017-314050

[11] de Wit DF, Hanssen NMJ, Wortelboer K, Herrema H, Rampanelli E, Nieuwdorp M (2023) Evidence for the contribution of the gut microbiome to obesity and its reversal. Sci Transl Med 15(723):eadg2773 37992156 10.1126/scitranslmed.adg2773

[12] Van Hul M, Cani PD (2023) The gut microbiota in obesity and weight management: microbes as friends or foe? Nat Reviews Endocrinol 19(5):258–271 10.1038/s41574-022-00794-0 36650295

[13] Kemp JVA, Kumar V, Saleem A, Hashman G, Hussain M, Taylor VH (2023) Examining associations between women’s Mental Health and obesity. Psychiatr Clin North Am 46(3):539–549 37500249 10.1016/j.psc.2023.04.009

[14] Bromet E, Andrade LH, Hwang I, Sampson NA, Alonso J, De Girolamo G, De Graaf R, Demyttenaere K, Hu C, Iwata N (2011) Cross-national epidemiology of DSM-IV major depressive episode. BMC Med 9:1–16 21791035 10.1186/1741-7015-9-90 PMC3163615

[15] Radjabzadeh D, Bosch JA, Uitterlinden AG, Zwinderman AH, Ikram MA, van Meurs JB, Luik AI, Nieuwdorp M, Lok A, van Duijn CM (2022) Gut microbiome-wide association study of depressive symptoms. Nat Commun 13(1):7128 36473852 10.1038/s41467-022-34502-3 PMC9726982

[16] (WHO) WHO, Depression, WHO (2024); [https://www.who.int/health-topics/depression#tab=tab_2

[17] Ferrari S, Mulè S, Parini F, Galla R, Ruga S, Rosso G, Brovero A, Molinari C, Uberti F (2024) The influence of the gut-brain axis on anxiety and depression: a review of the literature on the use of probiotics. J Traditional Complement Med 10.1016/j.jtcme.2024.03.011 PMC11069002 38707924

[18] Luqman A, He M, Hassan A, Ullah M, Zhang L, Rashid Khan M, Din AU, Ullah K, Wang W, Wang G (2024) Mood and microbes: a comprehensive review of intestinal microbiota’s impact on depression. Front Psychiatry 15 10.3389/fpsyt.2024.1295766 PMC10884216 38404464

[19] Turecki G, Brent DA, Gunnell D, O’Connor RC, Oquendo MA, Pirkis J, Stanley BH (2019) Suicide and suicide risk. Nat Reviews Disease Primers 5(1):74 31649257 10.1038/s41572-019-0121-0

[20] Ley RE, Bäckhed F, Turnbaugh P, Lozupone CA, Knight RD, Gordon JI (2005) Obesity alters gut microbial ecology. Proceedings of the national academy of sciences 102(31):11070-5 10.1073/pnas.0504978102 PMC1176910 16033867

[21] Ryan PM, Patterson E, Carafa I, Mandal R, Wishart DS, Dinan TG, Cryan JF, Tuohy KM, Stanton C, Ross RP (2020) Metformin and Dipeptidyl Peptidase-4 inhibitor differentially modulate the intestinal microbiota and plasma metabolome of metabolically dysfunctional mice. Can J Diabetes 44(2):146–55e2 31445961 10.1016/j.jcjd.2019.05.008

[22] Rigoulet M, Bouchez CL, Paumard P, Ransac S, Cuvellier S, Duvezin-Caubet S, Mazat JP, Devin A (2020) Cell energy metabolism: an update. Biochim et Biophys Acta (BBA)-Bioenergetics 1861(11):148276 32717222 10.1016/j.bbabio.2020.148276

[23] Galgani J, Ravussin E (2008) Energy metabolism, fuel selection and body weight regulation. Int J Obes 32(7):S109–S19 10.1038/ijo.2008.246 PMC2897177 19136979

[24] Hjorth MF, Roager HM, Larsen TM, Poulsen S, Licht TR, Bahl MI, Zohar Y, Astrup A (2018) Pre-treatment microbial Prevotella-to-Bacteroides ratio, determines body fat loss success during a 6-month randomized controlled diet intervention. Int J Obes 42(3):580–583 10.1038/ijo.2017.220 PMC5880576 28883543

[25] Hjorth MF, Blædel T, Bendtsen LQ, Lorenzen JK, Holm JB, Kiilerich P, Roager HM, Kristiansen K, Larsen LH, Astrup A (2019) Prevotella-to-Bacteroides ratio predicts body weight and fat loss success on 24-week diets varying in macronutrient composition and dietary fiber: results from a post-hoc analysis. Int J Obes 43(1):149–157 10.1038/s41366-018-0093-2 PMC6331389 29777234

[26] Behzadi P, Kim C-H, Pawlak EA, Algammal A (2023) The innate and adaptive immune system in human urinary system. Front Immunol 14:1294869 37876936 10.3389/fimmu.2023.1294869 PMC10593411

[27] Wang M, Song Z, Lai S, Tang F, Dou L, Yang F (2024) Depression-associated gut microbes, metabolites and clinical trials. Front Microbiol 15:1292004 38357350 10.3389/fmicb.2024.1292004 PMC10864537

[28] McGuinness A, Davis JA, Dawson S, Loughman A, Collier F, O’hely M, Simpson C, Green J, Marx W, Hair C (2022) A systematic review of gut microbiota composition in observational studies of major depressive disorder, bipolar disorder and schizophrenia. Mol Psychiatry 27(4):1920–1935 35194166 10.1038/s41380-022-01456-3 PMC9126816

[29] Zang Y, Lai X, Li C, Ding D, Wang Y, Zhu Y (2023) The role of gut microbiota in various Neurological and Psychiatric Disorders-An evidence mapping based on quantified evidence. Mediat Inflamm 2023:5127157 10.1155/2023/5127157 PMC9936509 36816743

[30] George F, Daniel C, Thomas M, Singer E, Guilbaud A, Tessier FJ, Revol-Junelles A-M, Borges F, Foligné B (2018) Occurrence and dynamism of lactic acid bacteria in distinct ecological niches: a multifaceted functional health perspective. Front Microbiol 9:2899 30538693 10.3389/fmicb.2018.02899 PMC6277688

[31] Giménez-Palomo A, Dodd S, Anmella G, Carvalho AF, Scaini G, Quevedo J, Pacchiarotti I, Vieta E, Berk M (2021) The role of mitochondria in mood disorders: from physiology to pathophysiology and to treatment. Front Psychiatry 12:546801 34295268 10.3389/fpsyt.2021.546801 PMC8291901

[32] Larrea A, Sánchez-Sánchez L, Diez-Martin E, Elexpe A, Torrecilla M, Astigarraga E, Barreda-Gómez G (2024) Mitochondrial metabolism in major depressive disorder: from early diagnosis to Emerging Treatment options. J Clin Med 13(6):1727 38541952 10.3390/jcm13061727 PMC10971738

[33] Steffen J, Focken N, Çalışkan G (2024) Recognizing depression as an inflammatory disease: the search for endotypes. Am J Physiol Cell Physiol 327(1):C205–c12 38826138 10.1152/ajpcell.00246.2024

[34] Gardiner JV, Jayasena CN, Bloom SR (2008) Gut hormones: a weight off your mind. J Neuroendocrinol 20(6):834–841 18601707 10.1111/j.1365-2826.2008.01729.x

[35] Steptoe A, Frank P (2023) Obesity and psychological distress. Philosophical Trans Royal Soc Lond Ser B Biol Sci 378(1888):20220225 10.1098/rstb.2022.0225 PMC10475872 37661745

[36] Song EJ, Shin NR, Jeon S, Nam YD, Kim H (2022) Lorcaserin and phentermine exert anti-obesity effects with modulation of the gut microbiota. Front Microbiol 13:1109651 36687627 10.3389/fmicb.2022.1109651 PMC9849812

[37] Pati B, Sendh S, Sahu B, Pani S, Jena N, Bal NC (2023) Recent advancements in pharmacological strategies to modulate energy balance for combating obesity. RSC Med Chem 14(8):1429–1445 37593583 10.1039/d3md00107e PMC10429841

[38] Lee CJ, Sears CL, Maruthur N (2020) Gut microbiome and its role in obesity and insulin resistance. Ann N Y Acad Sci 1461(1):37–52 31087391 10.1111/nyas.14107

[39] Wu L, Ye S, Deng X, Fu Z, Li J, Yang C (2024) Conjugated linoleic acid ameliorates high Fat-Induced insulin resistance via regulating gut microbiota-host metabolic and immunomodulatory interactions. Nutrients; 16(8) 10.3390/nu16081133 PMC11053735 38674824

[40] Shi Q, Wang Y, Hao Q, Vandvik PO, Guyatt G, Li J, Chen Z, Xu S, Shen Y, Ge L, Sun F, Li L, Yu J, Nong K, Zou X, Zhu S, Wang C, Zhang S, Qiao Z, Jian Z, Li Y, Zhang X, Chen K, Qu F, Wu Y, He Y, Tian H, Li S (2024) Pharmacotherapy for adults with overweight and obesity: a systematic review and network meta-analysis of randomised controlled trials. Lancet (London England) 403(10434):e21–e31 38582569 10.1016/S0140-6736(24)00351-9

[41] Boggio Marzet C, Burgos F, Del Compare M, Gerold I, Tabacco O, Vinderola G (2022) Approach to probiotics in pediatrics: the role of Lactobacillus rhamnosus GG. Arch Argentinos De Pediatria 120(1):e1–e7 10.5546/aap.2022.eng.e1 35068121

[42] Mills S, Yang B, Smith GJ, Stanton C, Ross RP (2023) Efficacy of Bifidobacterium longum alone or in multi-strain probiotic formulations during early life and beyond. Gut Microbes 15(1):2186098 36896934 10.1080/19490976.2023.2186098 PMC10012958

[43] Davies M, Færch L, Jeppesen OK, Pakseresht A, Pedersen SD, Perreault L, Rosenstock J, Shimomura I, Viljoen A, Wadden TA, Lingvay I (2021) Semaglutide 2·4 mg once a week in adults with overweight or obesity, and type 2 diabetes (STEP 2): a randomised, double-blind, double-dummy, placebo-controlled, phase 3 trial. Lancet (London England) 397(10278):971–984 33667417 10.1016/S0140-6736(21)00213-0

[44] Rubino D, Abrahamsson N, Davies M, Hesse D, Greenway FL, Jensen C, Lingvay I, Mosenzon O, Rosenstock J, Rubio MA, Rudofsky G, Tadayon S, Wadden TA, Dicker D (2021) Effect of continued Weekly Subcutaneous Semaglutide vs Placebo on Weight loss maintenance in adults with overweight or obesity: the STEP 4 Randomized Clinical Trial. JAMA 325(14):1414–1425 33755728 10.1001/jama.2021.3224 PMC7988425

[45] Mao T, Zhang C, Yang S, Bi Y, Li M, Yu J (2024) Semaglutide alters gut microbiota and improves NAFLD in db/db mice. Biochem Biophys Res Commun 710:149882 38583231 10.1016/j.bbrc.2024.149882

[46] Xiong C, Wu J, Ma Y, Li N, Wang X, Li Y, Ding X (2024) Effects of Glucagon-Like Peptide-1 receptor agonists on Gut Microbiota in Dehydroepiandrosterone-Induced Polycystic Ovary Syndrome mice: compared evaluation of Liraglutide and Semaglutide intervention. Diabetes Metabolic Syndrome Obesity: Targets Therapy 17:865–880 38406269 10.2147/DMSO.S451129 PMC10894520

[47] de Paiva IHR, da Silva RS, Mendonça IP, de Souza JRB, Peixoto CA (2024) Semaglutide attenuates anxious and depressive-like behaviors and reverses the cognitive impairment in a type 2 diabetes Mellitus Mouse Model Via the Microbiota-Gut-Brain Axis. J Neuroimmune Pharmacology: Official J Soc NeuroImmune Pharmacol 19(1):36 10.1007/s11481-024-10142-w 39042202

[48] Ying X, Rongjiong Z, Kahaer M, Chunhui J, Wulasihan M (2023) Therapeutic efficacy of liraglutide versus metformin in modulating the gut microbiota for treating type 2 diabetes mellitus complicated with nonalcoholic fatty liver disease. Front Microbiol 14:1088187 36778868 10.3389/fmicb.2023.1088187 PMC9909237

[49] Tobaiqy M, Elkout H (2024) Psychiatric adverse events associated with semaglutide, liraglutide and tirzepatide: a pharmacovigilance analysis of individual case safety reports submitted to the EudraVigilance database. Int J Clin Pharm 46(2):488–495 38265519 10.1007/s11096-023-01694-7 PMC10960895

[50] Tagliapietra GA, Cantrell MA, Lund BC (2024) Glucagon-like peptide receptor agonists and risk for depression. Prim Care Diabetes 10.1016/j.pcd.2024.05.005 38852027

[51] Arillotta D, Floresta G, Guirguis A, Corkery JM, Catalani V, Martinotti G, Sensi SL, Schifano F (2023) GLP-1 receptor agonists and related Mental Health issues; insights from a range of Social Media platforms using a mixed-methods Approach. Brain Sci.; 13(11) 10.3390/brainsci13111503 PMC10669484 38002464

[52] Mueller NT, Differding MK, Zhang M, Maruthur NM, Juraschek SP, Miller ER 3rd, Appel LJ, Yeh HC (2021) Metformin affects gut microbiome composition and function and circulating short-chain fatty acids: a Randomized Trial. Diabetes Care 44(7):1462–1471 34006565 10.2337/dc20-2257 PMC8323185

[53] Rosell-Díaz M, Petit-Gay A, Molas-Prat C, Gallardo-Nuell L, Ramió-Torrentà L, Garre-Olmo J, Pérez-Brocal V, Moya A, Jové M, Pamplona R, Puig J, Ramos R, Bäckhed F, Mayneris-Perxachs J, Fernández-Real JM (2024) Metformin-induced changes in the gut microbiome and plasma metabolome are associated with cognition in men. Metab Clin Exp 157:155941 38871078 10.1016/j.metabol.2024.155941

[54] Petakh P, Kobyliak N, Kamyshnyi A (2023) Gut microbiota in patients with COVID-19 and type 2 diabetes: a culture-based method. Front Cell Infect Microbiol 13:1142578 36844398 10.3389/fcimb.2023.1142578 PMC9947359

[55] Kessing LV, Rytgaard HC, Ekstrøm CT, Knop FK, Berk M, Gerds TA (2020) Antidiabetes agents and Incident Depression: a Nationwide Population-based study. Diabetes Care 43(12):3050–3060 32978179 10.2337/dc20-1561

[56] Yu H, Yang R, Wu J, Wang S, Qin X, Wu T, Hu Y, Wu Y (2022) Association of metformin and depression in patients with type 2 diabetes. J Affect Disord 318:380–385 36108876 10.1016/j.jad.2022.09.015

[57] Raineri S, Sherriff JA, Thompson KSJ, Jones H, Pfluger PT, Ilott NE, Mellor J (2022) Pharmacologically induced weight loss is associated with distinct gut microbiome changes in obese rats. BMC Microbiol 22(1):91 35392807 10.1186/s12866-022-02494-1 PMC8988407

[58] Uehira Y, Ueno H, Miyamoto J, Kimura I, Ishizawa Y, Iijima H, Muroga S, Fujita T, Sakai S, Samukawa Y, Tanaka Y, Murayama S, Sakoda H, Nakazato M (2023) Impact of the lipase inhibitor orlistat on the human gut microbiota. Obes Res Clin Pract 17(5):411–420 37679239 10.1016/j.orcp.2023.08.005

[59] Thai K, Taylor MW, Fernandes T, Akinade EA, Campbell SL (2023) Topiramate alters the gut microbiome to aid in its anti-seizure effect. Front Microbiol 14:1242856 37942078 10.3389/fmicb.2023.1242856 PMC10629356

[60] Deng L, Yang Y, Xu G (2022) Empagliflozin ameliorates type 2 diabetes mellitus-related diabetic nephropathy via altering the gut microbiota. Biochim et Biophys acta Mol cell Biology Lipids 1867(12):159234 10.1016/j.bbalip.2022.159234 36185030

[61] Grilo CM, Lydecker JA, Fineberg SK, Moreno JO, Ivezaj V, Gueorguieva R (2022) Naltrexone-bupropion and behavior therapy, alone and combined, for binge-eating disorder: Randomized double-blind placebo-controlled trial. Am J Psychiatry 179(12):927–937 36285406 10.1176/appi.ajp.20220267 PMC9722598

[62] Grilo CM, Kerrigan SG, Lydecker JA, White MA (2021) Physical activity changes during behavioral weight loss treatment by Latinx patients with obesity with and without binge eating disorder. Obes (Silver Spring Md) 29(12):2026–2034 10.1002/oby.23278 PMC8612949 34582624

[63] Kolotkin RL, Gadde KM, Peterson CA, Crosby RD (2016) Health-related quality of life in two randomized controlled trials of phentermine/topiramate for obesity: what mediates improvement? Qual life Research: Int J Qual life Aspects Treat care Rehabilitation 25(5):1237–1244 10.1007/s11136-015-1153-x 26446094

[64] Şahin S, Haliloğlu Ö, Polat Korkmaz Ö, Durcan E, Rekalı Şahin H, Yumuk VD, Damcı T, İlkova HM, Oşar Siva Z (2020) Does treatment with sodium-glucose co-transporter-2 inhibitors have an effect on sleep quality, quality of life, and anxiety levels in people with type 2 diabetes mellitus? Turk J Med Sci 51(2):735–742 33356033 10.3906/sag-2008-37 PMC8203126

[65] He L, Fu Y, Tian Y, Wang X, Zhou X, Ding RB, Qi X, Bao J (2023) Antidepressants as Autophagy modulators for Cancer Therapy. Molecules; 28(22) 10.3390/molecules28227594 PMC10673223 38005316

[66] Chen T, Cheng L, Ma J, Yuan J, Pi C, Xiong L, Chen J, Liu H, Tang J, Zhong Y, Zhang X, Liu Z, Zuo Y, Shen H, Wei Y, Zhao L (2023) Molecular mechanisms of rapid-acting antidepressants: new perspectives for developing antidepressants. Pharmacol Res 194:106837 37379962 10.1016/j.phrs.2023.106837

[67] Rukavishnikov G, Leonova L, Kasyanov E, Leonov V, Neznanov N, Mazo G (2023) Antimicrobial activity of antidepressants on normal gut microbiota: results of the in vitro study. Front Behav Neurosci 17:1132127 37035624 10.3389/fnbeh.2023.1132127 PMC10073483

[68] Xu F, Xie Q, Kuang W, Dong Z (2023) Interactions between antidepressants and Intestinal Microbiota. Neurotherapeutics: J Am Soc Experimental Neurother 20(2):359–371 10.1007/s13311-023-01362-8 PMC10121977 36881351

