# Supplementary material for: The interplay of gut microbiota, obesity, and depression: insights and interventions
Source: Cell Mol Life Sci. 2024 Oct 30;81(1):443. doi: 10.1007/s00018-024-05476-w (PMC11525354; doi:10.1007/s00018-024-05476-w)
Supplement: Supplementary file 1 — Supplementary Material 1 [file 18_2024_5476_MOESM1_ESM.docx]

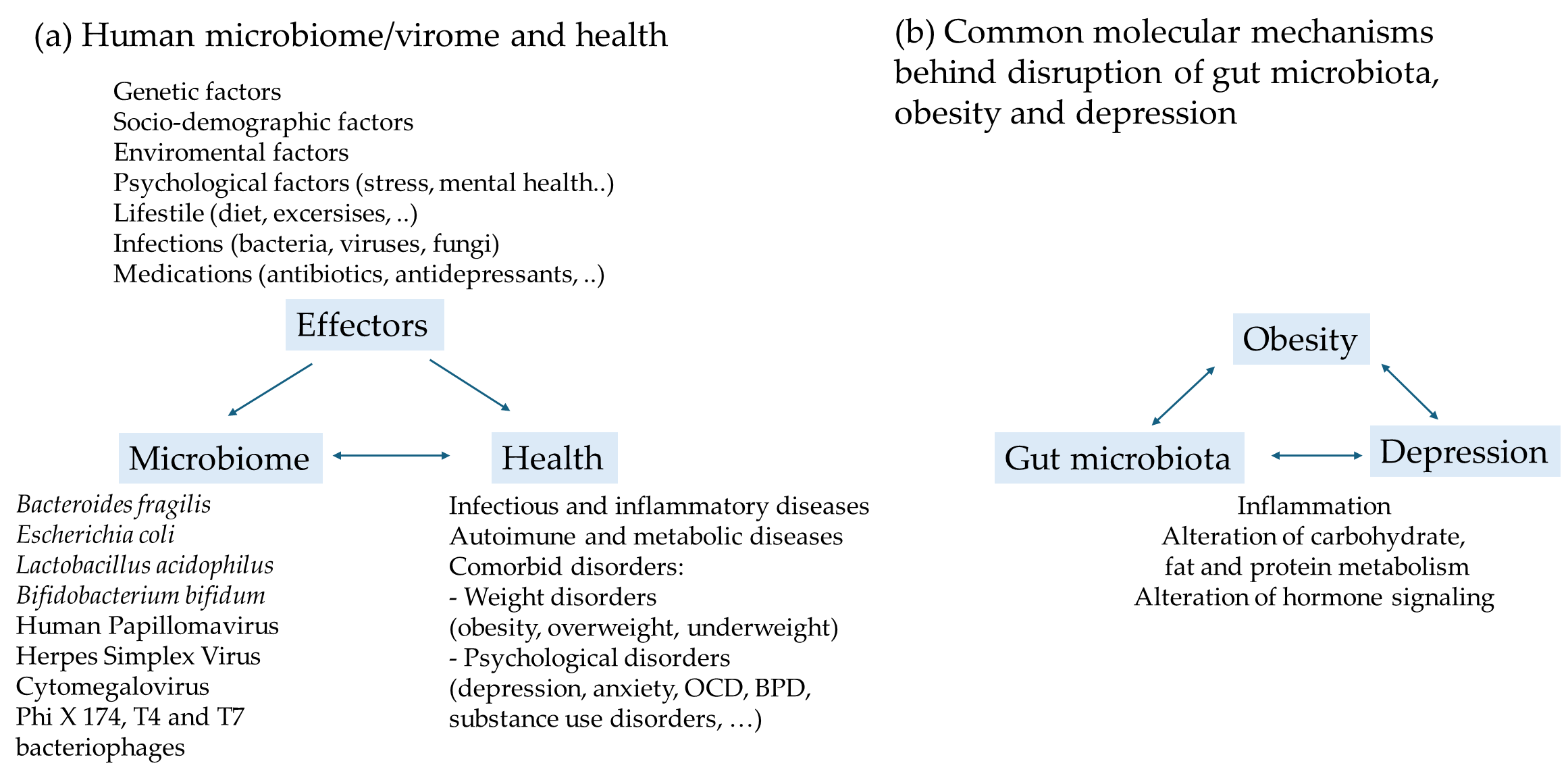


**Figure S1.** The Interplay of Gut Microbiota, Obesity, and Depression. (a) Relationship between human microbiome and health. The diagram depicts the relationship between human health and the microbiome, mediated by various effectors such as genetic, socio-demographic, environmental, psychological factors, lifestyle, infections, and medications. (b). The interconnection between gut microbiota, obesity and depression. These conditions influence each other bidirectionally, creating a complex interplay that impacts overall health.

**Table S1**. Approaches for disruption of imbalanced gut microbiota, obesity and depression.

|  | **Disruption of gut microbiota** | **Obesity** | **Depression** |
| --- | --- | --- | --- |
| **Lifestyle changes** | Diet  Stress management Regular exercise  Avoidance of harmful substances | Diet  Stress management  Regular exercise  Avoidance of harmful substances | Diet  Stress management  Regular exercise  Avoidance of harmful substances  Sleep |
| **Medical interventions** | Antibiotics  Probiotics  Fecal Microbiota Transplant | Weight loss drugs  Anti-diabetic drugs  Anti-hypertension drugs | Antidepressants |
| **Surgical options** |  | Gastric bypass, sleeve gastrectomy, or adjustable banding |  |
| **Psychotherapy** | Behavioral therapy | Behavioral therapy | Behavioral Therapy  Interpersonal Therapy Psychodynamic Therapy |

**Table S2**. Impact of Obesity Treatments on Gut Microbiome and Mental Health in Animal Studies

| **Author, year** | **Type of animals** | **Impact on gut microbiome and mental health** |
| --- | --- | --- |
| **GLP-1 receptor agonists** | | |
| Mao et al, 2024 ^1^ | Db/db mice | **Semaglutide** caused significant alterations observed in the genera *Alloprevotella*, *Alistipes,* *Ligilactobacillus*, and *Lactobacillus*. |
| Liu et al, 2020 ^2^ | Db/db mice | **Liraglutide** increased the abundance of *Akkermansia* **↑**, *Romboutsia* **↑**, and *Bacteroidales* **↑,** it decreased the abundance of *Klebsiella* ↓, *Anaerotruncus* ↓, *Bacteroides* ↓*, Lachnospiraceae* ↓*, Ruminiclostridium* ↓*, Ruminococcaceae* ↓, and *Desulfovibrio* ↓ |
| Wang et al, 2016 ^3^ | Germ-free mice | 13 phylotypes **↑** increased and 20 phylotypes ↓ decreased under **liraglutide** administration |
| Xiong et al, 2024 ^4^ | Female C57BL/6J mice | **Liraglutide** increased the *Bacillota*-to-*Bacteroidota* ratio **↑**. **Semaglutide** raised *Helicobacter* abundance **↑** |
| de Paiva et al, 2024 ^5^ | Diabetic mice | **Semaglutide** treatment increased *Bacteroidetes* **↑***, Bacteroides acidifaciens* **↑**, and *Blautia coccoides* **↑** in the gut microbiota. **Semaglutide** reduced depressive-like behaviors. |
| Sharma et al, 2015 ^6^ | Rats | **Liraglutide** reverses depression and metabolic issues from long-term antipsychotic use**.** |
| **Metformin** | | |
| Zhang et al, 2019 ^7^ | Db/db mice | **Metformin** enhanced SCFA-producing bacteria (*Butyricimonas* **↑***, Coprococcus* **↑***, Ruminococcus* **↑**) and reduced opportunistic pathogens (*Prevotella* ↓*, Proteus* ↓). |
| Chen et al, 2023 ^8^ | Specific pathogen-free male Sprague-Dawley rats | **Metformin** modified the abundance of *Atopobiaceae, Brevibacterium, Christensenellaceae, Coriobacteriales, Papillibacter, Pygmaiobacter,* and *Rikenellaceae* RC9. |
| Yang et al, 2022 ^9^ | Wild-type male C57BL/6 mice | **Metformin**'s effects on psychiatric symptoms involve gut microbiota metabolites. |
| Zhu et al, 2023 ^10^ | Aged mice | **Metformin** increases *Akkermansia muciniphila* **↑**, improving cognitive function by reducing interleukin-6. |
| Broadfield et al, 2022 ^11^ | High-fat diet-fed mice | **Metformin** increased *Alistipes, Lachnospiraceae* **↑**, and *Ruminococcaceae* **↑***.* |
| Shin et al, 2014 ^12^ | Diet-induced obese mice | **Metformin** increases *Akkermansia* spp. **↑**, improving glucose homeostasis in obese mice. |
| Ryan et al, 2020 ^13^ | C57BL/6 mice | **Metformin** decreased *Firmicutes/Bacteroidetes* ratios ↓ and increased *Akkermansia* **↑***, Parabacteroides* **↑**, and *Christensenella* **↑***.* |
| Lyu et al, 2022 ^14^ | Db/db mice | **Metformin** and berberine co-treatment significantly altered *Proteobacteria* and *Verrucomicrobia* |
| Lin et al, 2022 ^15^ | 13-month-old apoE3-targeted replacement (TR) and apoE4-TR mice | **Metformin** improves depression by enhancing glucose metabolism and mitochondrial biogenesis. |
| Hao et al, 2021 ^16^ | Rats | **Metformin** reduces corticosterone-induced metabolic and depressive effects via glucose metabolism. |
| Hammad et al, 2021 ^17^ | Wistar male rats | **Metformin** reduces oxandrolone-induced depression by modulating IL-1β, IL-6, IL-10, and TNF-α. |
| Chen et al, 2020 ^18^ | LPS-treated mice | **Metformin** alleviates depressive-like behaviors and enhances glutamatergic transmission. |
| Fang et al, 2020 ^19^ | C57BL/6J mice | **Metformin** reduces stress-induced depression by enhancing BDNF via AMPK/CREB. |
| Du et al, 2020 ^20^ | Mice | **Metformin** alleviates depressive-like symptoms by inhibiting NF-κB-NLRP3 inflammation. |
| **Bupropion-naltrexone** | | |
| Raineri et al, 2022 ^21^ | Wistar rats | **Bupropion** and **naltrexone** altered gut microbiome beta diversity. |
| **Topiramate** | | |
| Thai et al, 2023 ^22^ | C57BL/6J mice | **Topiramate** elevated *Lactobacillus johnsonii* **↑** levels significantly. |
| Miao et al, 2022 ^23^ | Specific pathogen-free male Sprague–Dawley rats | **Topiramate** treatment reduced *Lactobacillus* genus ↓. |
| **SGLT2 inhibitors** | | |
| Deng et al, 2022 ^24^ | Male C57BL/6J mice | **Sodium-glucose co-transporter 2** administration altered *Oscillibacter, Bacteroides,* and *Odoribacter.* |

**Table S3.** Impact of Obesity Treatments on Gut Microbiome and Mental Health in Human Studies

| **Author, year** | **Study design** | **Participants** | **Number of participants** | **Impact on gut microbiome and mental health** |
| --- | --- | --- | --- | --- |
| Wium-Andersen et al, 2022 ^25^ | A population-based cohort and nested case-control study | Patients with T2DM | n=116,699 | Low doses of **metformin, DPP-4 inhibitors, GLP-1 analogs,** and **SGLT-2 inhibitors** reduce depression risk in diabetes patients. |
| **GLP-1 receptor agonists** | | | | |
| Ying et al, 2023 ^26^ | Randomized trials | Patients with T2DM complicated with NAFLD | n=15 | **Liraglutide** significantly elevated the relative abundances of *Bacteroidetes* **↑***,* *Proteobacteria* **↑***,* and *Bacilli* **↑** |
| Tobaiqy et al, 2024 ^27^ | Retrospective analysis | Patients receiving GLP-1 receptor agonists | n=31,444 | Psychiatric adverse events constituted a mere 1.2% of the aggregate reports concerning **semaglutide, liraglutide, and tirzepatide**. |
| Li et al, 2023 ^28^ | A case report | A middle-aged man without a prior history of depression, a middle-aged woman with a history of recurrent depressive disorder | n=2 | **Semaglutide** treatment led to depressive symptoms in both patients within a month. |
| Tagliapietra et al, 2024 ^29^ | Retrospective cohort study | Veterans Health Administration patients | n=34,130 | In patients on **GLP-1 receptor agonists**, depression incidence was 7.7%. |
| Nguyen et al, 2024 ^30^ | Mendelian randomization study | T2DM and obesity patients | N/A | MR analyses showed no causal link between higher endogenous **GLP-1R agonism** and suicide risk. |
| Manoharan et al, 2024 ^31^ | A case report | 54-year-old female patient | n=1 | A case report noted mood changes with **semaglutide** in depressive patients. |
| McIntyre et al, 2024 ^32^ | Meta-analysis | Patients receiving liraglutide and semaglutide | N/A | **Semaglutide** and **liraglutide** showed disproportionate reporting of suicidal ideation, but not suicidal behaviors or deaths. |
| Arillotta et al, 2023 ^33^ | Retrospective analysis | Patients receiving semaglutide | n=43,710 | Recent reports highlighted the potential risks of **semaglutide,** with 204 out of 43,710 patient posts specifically mentioning depression. |
| **Metformin** | | | | |
| Mueller et al, 2021 ^34^ | Randomized trials | Overweight/obese adults | n=121 | **Metformin** treatment increased *Escherichia coli* **↑** and *Ruminococcus torques* **↑** while decreasing *Intestinibacter bartlettii* ↓ and the genus *Roseburia* ↓ |
| Rosell-Diaz et al, 2024 ^35^ | Randomized, double-blind, parallel-group, randomized pilot study | Patients with T2DM | n=373 | **Metformin** treatment was positively associated with the genera *Escherichia* **↑** and *Akkermansia* **↑**, but negatively associated with the genus *Romboutsia* ↓*.* |
| Lee et al, 2021 ^36^ | Open-label and single-arm clinical trial | Healthy Korean | n=20 | **Metformin** administration altered the relative abundances of *Escherichia, Romboutsia, Intestinibacter*, and *Clostridium*. |
| Molina-Vega et al, 2022 ^37^ | Randomized trials | Women with gestational diabetes mellitus | n=30 | **Metformin** treatment resulted in a decline in *Firmicutes* ↓ and *Peptostreptococcaceae* ↓, and an increase in *Proteobacteria* **↑** and E*nterobacteriaceae* **↑**. |
| Petakh et al, 2023 ^38^ | Randomized trials | Patients with COVID-19 and T2DM | n=128 | **Metformin**-treated patients showed increased *Bacteroides* spp. **↑** and *Lactobacillus* spp. **↑**, but decreased *Enterococcus* ↓ and *Clostridium* ↓ |
| Calkin et al, 2022 ^39^ | Randomized, Quadruple-Masked, Placebo-Controlled Clinical Trial | Patients with treatment-resistant bipolar depression | n=45 | **Metformin** may help treat resistant bipolar depression. |
| Yu et al, 2022 ^40^ | Randomized trials | Patients with T2DM | n=517,554 | **Metformin** reduces depression risk compared to other oral hypoglycemic agents. |
| Kessing et al, 2020 ^41^ | A Nationwide Population-Based Study | Patients with T2DM | n=319,917 | **Metformin** has a beneficial effect on depression rates. |
| **Orlistat** | | | | |
| Uehira et al, 2023 ^42^ | Randomized trials | Obese men | n=14 | After eight weeks of **orlistat** treatment, participants showed reduced *Firmicutes* ↓, increased *Bacteroidetes* **↑**, and elevated *Lactobacillus* levels **↑**. |
| Grilo et al, 2022 ^43^ | Randomized controlled trial | Patients with obesity | n=79 | **Orlistat** treatment in Latinx patients with obesity was associated with reductions in depression. |
| **Phentermine-topiramate** | | | | |
| Homola et al, 2018 ^44^ | A case report | 40-year-old Hispanic woman | n=1 | Venlafaxine with **phentermine/topiramate** may induce psychosis. |
| Kolotkin et al, 2016 | Randomized trials | Patients with obesity | n=2374 | **Phentermine/topiramate** reduces depressive symptoms in obese patients. |
| Jumaili et al, 2024 ^45^ | A case report | 40-year-old White mother of 4 with bipolar II disorder and morbid obesity | n=1 | **Phentermine** may induce hypomania in bipolar depression patients. |
| **SGLT2 inhibitors** | | | | |
| Kusunoki et al, 2023 ^46^ | Randomized trials | Patients with T2DM | n=36 | Treatment with an **SGLT2 inhibitor** significantly increased the prevalence of *Ruminococci* **↑** |
| Şahin ey al, 2020 ^47^ | Randomized trials | Patients with T2DM | n=97 | **SGLT2 inhibitors** improved quality of life in diabetes patients through weight loss. |
| Zandifar et al, 2024 ^48^ | Randomized controlled trial | Patients with moderate and severe depression | n=90 | The study demonstrates favorable outcomes with **empagliflozin** as adjunctive MDD treatment. |

References

1. Mao, T.; Zhang, C.; Yang, S.; Bi, Y.; Li, M.; Yu, J., Semaglutide alters gut microbiota and improves NAFLD in db/db mice. *Biochemical and biophysical research communications* **2024,** *710*, 149882.

2. Liu, Q.; Cai, B. Y.; Zhu, L. X.; Xin, X.; Wang, X.; An, Z. M.; Li, S.; Hu, Y. Y.; Feng, Q., Liraglutide modulates gut microbiome and attenuates nonalcoholic fatty liver in db/db mice. *Life sciences* **2020,** *261*, 118457.

3. Wang, L.; Li, P.; Tang, Z.; Yan, X.; Feng, B., Structural modulation of the gut microbiota and the relationship with body weight: compared evaluation of liraglutide and saxagliptin treatment. *Sci Rep* **2016,** *6*, 33251.

4. Xiong, C.; Wu, J.; Ma, Y.; Li, N.; Wang, X.; Li, Y.; Ding, X., Effects of Glucagon-Like Peptide-1 Receptor Agonists on Gut Microbiota in Dehydroepiandrosterone-Induced Polycystic Ovary Syndrome Mice: Compared Evaluation of Liraglutide and Semaglutide Intervention. *Diabetes, metabolic syndrome and obesity : targets and therapy* **2024,** *17*, 865-880.

5. de Paiva, I. H. R.; da Silva, R. S.; Mendonça, I. P.; de Souza, J. R. B.; Peixoto, C. A., Semaglutide Attenuates Anxious and Depressive-Like Behaviors and Reverses the Cognitive Impairment in a Type 2 Diabetes Mellitus Mouse Model Via the Microbiota-Gut-Brain Axis. *Journal of neuroimmune pharmacology : the official journal of the Society on NeuroImmune Pharmacology* **2024,** *19* (1), 36.

6. Sharma, A. N.; Ligade, S. S.; Sharma, J. N.; Shukla, P.; Elased, K. M.; Lucot, J. B., GLP-1 receptor agonist liraglutide reverses long-term atypical antipsychotic treatment associated behavioral depression and metabolic abnormalities in rats. *Metab Brain Dis* **2015,** *30* (2), 519-27.

7. Zhang, W.; Xu, J. H.; Yu, T.; Chen, Q. K., Effects of berberine and metformin on intestinal inflammation and gut microbiome composition in db/db mice. *Biomedicine & pharmacotherapy = Biomedecine & pharmacotherapie* **2019,** *118*, 109131.

8. Chen, D.; Xiong, J.; Chen, G.; Zhang, Z.; Liu, Y.; Xu, J.; Xu, H., Comparing the Influences of Metformin and Berberine on the Intestinal Microbiota of Rats With Nonalcoholic Steatohepatitis. *In vivo (Athens, Greece)* **2023,** *37* (5), 2105-2127.

9. Yang, J.; Zhang, Z.; Xie, Z.; Bai, L.; Xiong, P.; Chen, F.; Zhu, T.; Peng, Q.; Wu, H.; Zhou, Y.; Ma, Y.; Zhang, Y.; Chen, M.; Gao, J.; Tian, W.; Shi, K.; Du, Y.; Duan, Y.; Wang, H.; Xu, Y.; Kuang, Y. Q.; Zhu, M.; Yu, J.; Wang, K., Metformin modulates microbiota-derived inosine and ameliorates methamphetamine-induced anxiety and depression-like withdrawal symptoms in mice. *Biomedicine & pharmacotherapy = Biomedecine & pharmacotherapie* **2022,** *149*, 112837.

10. Zhu, X.; Shen, J.; Feng, S.; Huang, C.; Wang, H.; Huo, F.; Liu, H., Akkermansia muciniphila, which is enriched in the gut microbiota by metformin, improves cognitive function in aged mice by reducing the proinflammatory cytokine interleukin-6. *Microbiome* **2023,** *11* (1), 120.

11. Broadfield, L. A.; Saigal, A.; Szamosi, J. C.; Hammill, J. A.; Bezverbnaya, K.; Wang, D.; Gautam, J.; Tsakiridis, E. E.; Di Pastena, F.; McNicol, J.; Wu, J.; Syed, S.; Lally, J. S. V.; Raphenya, A. R.; Blouin, M. J.; Pollak, M.; Sacconi, A.; Blandino, G.; McArthur, A. G.; Schertzer, J. D.; Surette, M. G.; Collins, S. M.; Bramson, J. L.; Muti, P.; Tsakiridis, T.; Steinberg, G. R., Metformin-induced reductions in tumor growth involves modulation of the gut microbiome. *Mol Metab* **2022,** *61*, 101498.

12. Shin, N. R.; Lee, J. C.; Lee, H. Y.; Kim, M. S.; Whon, T. W.; Lee, M. S.; Bae, J. W., An increase in the Akkermansia spp. population induced by metformin treatment improves glucose homeostasis in diet-induced obese mice. *Gut* **2014,** *63* (5), 727-35.

13. Ryan, P. M.; Patterson, E.; Carafa, I.; Mandal, R.; Wishart, D. S.; Dinan, T. G.; Cryan, J. F.; Tuohy, K. M.; Stanton, C.; Ross, R. P., Metformin and Dipeptidyl Peptidase-4 Inhibitor Differentially Modulate the Intestinal Microbiota and Plasma Metabolome of Metabolically Dysfunctional Mice. *Canadian journal of diabetes* **2020,** *44* (2), 146-155.e2.

14. Lyu, Y.; Li, D.; Yuan, X.; Li, Z.; Zhang, J.; Ming, X.; Shaw, P. C.; Zhang, C.; Kong, A. P. S.; Zuo, Z., Effects of combination treatment with metformin and berberine on hypoglycemic activity and gut microbiota modulation in db/db mice. *Phytomedicine : international journal of phytotherapy and phytopharmacology* **2022,** *101*, 154099.

15. Lin, Y.; Dai, X.; Zhang, J.; Chen, X., Metformin alleviates the depression-like behaviors of elderly apoE4 mice via improving glucose metabolism and mitochondrial biogenesis. *Behavioural brain research* **2022,** *423*, 113772.

16. Hao, Y.; Tong, Y.; Guo, Y.; Lang, X.; Huang, X.; Xie, X.; Guan, Y.; Li, Z., Metformin Attenuates the Metabolic Disturbance and Depression-like Behaviors Induced by Corticosterone and Mediates the Glucose Metabolism Pathway. *Pharmacopsychiatry* **2021,** *54* (3), 131-141.

17. Hammad, A. M.; Ibrahim, Y. A.; Khdair, S. I.; Hall, F. S.; Alfaraj, M.; Jarrar, Y.; Abed, A. F., Metformin reduces oxandrolone- induced depression-like behavior in rats via modulating the expression of IL-1β, IL-6, IL-10 and TNF-α. *Behavioural brain research* **2021,** *414*, 113475.

18. Chen, J.; Zhou, T.; Guo, A. M.; Chen, W. B.; Lin, D.; Liu, Z. Y.; Fei, E. K., Metformin Ameliorates Lipopolysaccharide-Induced Depressive-Like Behaviors and Abnormal Glutamatergic Transmission. *Biology* **2020,** *9* (11).

19. Fang, W.; Zhang, J.; Hong, L.; Huang, W.; Dai, X.; Ye, Q.; Chen, X., Metformin ameliorates stress-induced depression-like behaviors via enhancing the expression of BDNF by activating AMPK/CREB-mediated histone acetylation. *J Affect Disord* **2020,** *260*, 302-313.

20. Du, R. W.; Bu, W. G., Metformin improves depressive-like symptoms in mice via inhibition of peripheral and central NF-κB-NLRP3 inflammation activation. *Experimental brain research* **2020,** *238* (11), 2549-2556.

21. Raineri, S.; Sherriff, J. A.; Thompson, K. S. J.; Jones, H.; Pfluger, P. T.; Ilott, N. E.; Mellor, J., Pharmacologically induced weight loss is associated with distinct gut microbiome changes in obese rats. *BMC microbiology* **2022,** *22* (1), 91.

22. Thai, K.; Taylor, M. W.; Fernandes, T.; Akinade, E. A.; Campbell, S. L., Topiramate alters the gut microbiome to aid in its anti-seizure effect. *Front Microbiol* **2023,** *14*, 1242856.

23. Miao, S.; Tang, W.; Li, H.; Li, B.; Yang, C.; Xie, W.; Wang, T.; Bai, W.; Gong, Z.; Dong, Z.; Yu, S., Repeated inflammatory dural stimulation-induced cephalic allodynia causes alteration of gut microbial composition in rats. *The journal of headache and pain* **2022,** *23* (1), 71.

24. Deng, L.; Yang, Y.; Xu, G., Empagliflozin ameliorates type 2 diabetes mellitus-related diabetic nephropathy via altering the gut microbiota. *Biochimica et biophysica acta. Molecular and cell biology of lipids* **2022,** *1867* (12), 159234.

25. Wium-Andersen, I. K.; Osler, M.; Jørgensen, M. B.; Rungby, J.; Wium-Andersen, M. K., Diabetes, antidiabetic medications and risk of depression - A population-based cohort and nested case-control study. *Psychoneuroendocrinology* **2022,** *140*, 105715.

26. Ying, X.; Rongjiong, Z.; Kahaer, M.; Chunhui, J.; Wulasihan, M., Therapeutic efficacy of liraglutide versus metformin in modulating the gut microbiota for treating type 2 diabetes mellitus complicated with nonalcoholic fatty liver disease. *Front Microbiol* **2023,** *14*, 1088187.

27. Tobaiqy, M.; Elkout, H., Psychiatric adverse events associated with semaglutide, liraglutide and tirzepatide: a pharmacovigilance analysis of individual case safety reports submitted to the EudraVigilance database. *International journal of clinical pharmacy* **2024,** *46* (2), 488-495.

28. Li, J. R.; Cao, J.; Wei, J.; Geng, W., Case Report: Semaglutide-associated depression: a report of two cases. *Front Psychiatry* **2023,** *14*, 1238353.

29. Tagliapietra, G. A.; Cantrell, M. A.; Lund, B. C., Glucagon-like peptide receptor agonists and risk for depression. *Primary care diabetes* **2024**.

30. Nguyen, A.; Smith, E.; Hashemy, H.; Agarwal, S. M.; Hahn, M. K.; Paterson, A. D.; Dash, S., Glucagon-like-peptide 1 receptor agonism and attempted suicide: A Mendelian randomisation study to assess a potential causal association. *Clinical obesity* **2024,** *14* (4), e12676.

31. Manoharan, S.; Madan, R., GLP-1 Agonists Can Affect Mood: A Case of Worsened Depression on Ozempic (Semaglutide). *Innovations in clinical neuroscience* **2024,** *21* (4-6), 25-26.

32. McIntyre, R. S.; Mansur, R. B.; Rosenblat, J. D.; Kwan, A. T. H., The association between glucagon-like peptide-1 receptor agonists (GLP-1 RAs) and suicidality: reports to the Food and Drug Administration Adverse Event Reporting System (FAERS). *Expert opinion on drug safety* **2024,** *23* (1), 47-55.

33. Arillotta, D.; Floresta, G.; Guirguis, A.; Corkery, J. M.; Catalani, V.; Martinotti, G.; Sensi, S. L.; Schifano, F., GLP-1 Receptor Agonists and Related Mental Health Issues; Insights from a Range of Social Media Platforms Using a Mixed-Methods Approach. *Brain sciences* **2023,** *13* (11).

34. Mueller, N. T.; Differding, M. K.; Zhang, M.; Maruthur, N. M.; Juraschek, S. P.; Miller, E. R., 3rd; Appel, L. J.; Yeh, H. C., Metformin Affects Gut Microbiome Composition and Function and Circulating Short-Chain Fatty Acids: A Randomized Trial. *Diabetes care* **2021,** *44* (7), 1462-1471.

35. Rosell-Díaz, M.; Petit-Gay, A.; Molas-Prat, C.; Gallardo-Nuell, L.; Ramió-Torrentà, L.; Garre-Olmo, J.; Pérez-Brocal, V.; Moya, A.; Jové, M.; Pamplona, R.; Puig, J.; Ramos, R.; Bäckhed, F.; Mayneris-Perxachs, J.; Fernández-Real, J. M., Metformin-induced changes in the gut microbiome and plasma metabolome are associated with cognition in men. *Metabolism: clinical and experimental* **2024,** *157*, 155941.

36. Lee, Y.; Kim, A. H.; Kim, E.; Lee, S.; Yu, K. S.; Jang, I. J.; Chung, J. Y.; Cho, J. Y., Changes in the gut microbiome influence the hypoglycemic effect of metformin through the altered metabolism of branched-chain and nonessential amino acids. *Diabetes research and clinical practice* **2021,** *178*, 108985.

37. Molina-Vega, M.; Picón-César, M. J.; Gutiérrez-Repiso, C.; Fernández-Valero, A.; Lima-Rubio, F.; González-Romero, S.; Moreno-Indias, I.; Tinahones, F. J., Metformin action over gut microbiota is related to weight and glycemic control in gestational diabetes mellitus: A randomized trial. *Biomedicine & pharmacotherapy = Biomedecine & pharmacotherapie* **2022,** *145*, 112465.

38. Petakh, P.; Kobyliak, N.; Kamyshnyi, A., Gut microbiota in patients with COVID-19 and type 2 diabetes: A culture-based method. *Frontiers in cellular and infection microbiology* **2023,** *13*, 1142578.

39. Calkin, C. V.; Chengappa, K. N. R.; Cairns, K.; Cookey, J.; Gannon, J.; Alda, M.; O'Donovan, C.; Reardon, C.; Sanches, M.; Růzicková, M., Treating Insulin Resistance With Metformin as a Strategy to Improve Clinical Outcomes in Treatment-Resistant Bipolar Depression (the TRIO-BD Study): A Randomized, Quadruple-Masked, Placebo-Controlled Clinical Trial. *The Journal of clinical psychiatry* **2022,** *83* (2).

40. Yu, H.; Yang, R.; Wu, J.; Wang, S.; Qin, X.; Wu, T.; Hu, Y.; Wu, Y., Association of metformin and depression in patients with type 2 diabetes. *J Affect Disord* **2022,** *318*, 380-385.

41. Kessing, L. V.; Rytgaard, H. C.; Ekstrøm, C. T.; Knop, F. K.; Berk, M.; Gerds, T. A., Antidiabetes Agents and Incident Depression: A Nationwide Population-Based Study. *Diabetes care* **2020,** *43* (12), 3050-3060.

42. Uehira, Y.; Ueno, H.; Miyamoto, J.; Kimura, I.; Ishizawa, Y.; Iijima, H.; Muroga, S.; Fujita, T.; Sakai, S.; Samukawa, Y.; Tanaka, Y.; Murayama, S.; Sakoda, H.; Nakazato, M., Impact of the lipase inhibitor orlistat on the human gut microbiota. *Obesity research & clinical practice* **2023,** *17* (5), 411-420.

43. Grilo, C. M.; Kerrigan, S. G.; Lydecker, J. A.; White, M. A., Physical activity changes during behavioral weight loss treatment by Latinx patients with obesity with and without binge eating disorder. *Obesity (Silver Spring, Md.)* **2021,** *29* (12), 2026-2034.

44. Homola, J.; Hieber, R., Combination of venlafaxine and phentermine/topiramate induced psychosis: A case report. *The mental health clinician* **2018,** *8* (2), 95-99.

45. Jumaili, W. A.; Gburi, N. A.; Jain, S., Weight Loss Medication Phentermine-Induced Hypomania in Bipolar Depression. *The primary care companion for CNS disorders* **2024,** *26* (1).

46. Kusunoki, M.; Hisano, F.; Matsuda, S. I.; Kusunoki, A.; Wakazono, N.; Tsutsumi, K.; Miyata, T., Effects of SGLT2 inhibitors on the intestinal bacterial flora in Japanese patients with type 2 diabetes mellitus. *Drug research* **2023,** *73* (7), 412-416.

47. Şahin, S.; Haliloğlu, Ö.; Polat Korkmaz, Ö.; Durcan, E.; Rekalı Şahin, H.; Yumuk, V. D.; Damcı, T.; İlkova, H. M.; Oşar Siva, Z., Does treatment with sodium-glucose co-transporter-2 inhibitors have an effect on sleep quality, quality of life, and anxiety levels in people with Type 2 diabetes mellitus? *Turkish journal of medical sciences* **2020,** *51* (2), 735-742.

48. Zandifar, A.; Panahi, M.; Badrfam, R.; Qorbani, M., Efficacy of empagliflozin as adjunctive therapy to citalopram in major depressive disorder: a randomized double-blind, placebo-controlled clinical trial. *BMC psychiatry* **2024,** *24* (1), 163.
